# Correlates of domain-specific sedentary behaviors and objectively assessed sedentary time among elementary school children

**DOI:** 10.1038/s41598-022-23410-7

**Published:** 2022-11-07

**Authors:** Mohammad Javad Koohsari, Koichiro Oka, Ai Shibata, Gavin R. McCormack, Tomoya Hanibuchi, Tomoki Nakaya, Kaori Ishii

**Affiliations:** 1 School of Knowledge Science, Japan Advanced Institute of Science and Technology, Nomi, Japan; 2 Faculty of Sport Sciences, Waseda University, Tokorozawa, Japan; 3 Faculty of Health and Sport Sciences, University of Tsukuba, Tsukuba, Japan; 4 Department of Community Health Sciences, Cumming School of Medicine, University of Calgary, Calgary, Canada; 5 Faculty of Kinesiology, University of Calgary, Calgary, Canada; 6 School of Architecture, Planning and Landscape, University of Calgary, Calgary, Canada; 7 Graduate School of Environmental Studies, Tohoku University, Sendai, Japan

**Keywords:** Health occupations, Risk factors

## Abstract

Understanding the correlates of sedentary behavior among children is essential in developing effective interventions to reduce sitting time in this vulnerable population. This study aimed to identify correlates of domain-specific sedentary behaviors and objectively assessed sedentary time among a sample of children in Japan. Data from 343 children (aged 6–12 years) living in Japan were used. Domain-specific sedentary behaviors were assessed using a questionnaire. Total sedentary time was estimated using hip-worn accelerometers. Twenty-two potential correlates across five categories (parental characteristics, household indoor environment, residential neighborhood environment, school environment, and school neighborhood environment) were included. Multivariable linear regression models were used to identify correlates of domain-specific sedentary behaviors and objectively assessed sedentary time. Eight correlates were significantly associated with children’s domain-specific sedentary behaviors: mother’s and father’s age, mother’s educational level, having a video/DVD recorder/player, having a video console, having a TV one’s own room, home’s Walk Score^®^, and pedestrian/cycling safety. No significant associations were found between potential correlates and accelerometer-based total sedentary time. These findings highlight that strategies to reduce children’s sedentary time should consider the context of these behaviors. For example, urban design attributes such as perceived pedestrian and cycling safety can be improved to reduce children’s car sitting time.

## Introduction

Sedentary behavior such as television viewing or computer use is an emerging risk factor for cardiometabolic health in children that is independent of physical activity levels^1^. For instance, a recent systematic review of fifty articles found that sedentary behavior was adversely associated with several cardiovascular risk factors, such as body adiposity, reduced level of high-density lipoprotein cholesterol, and elevated blood pressure, in children^2^. Another systematic review and meta-analysis found that reducing sedentary time was associated with reduced body mass index in school-aged children and youth^3^. Sedentary behavior is defined as "any waking behavior characterized by an energy expenditure ≤ 1.5 metabolic equivalents (METs) while in a sitting or reclining posture"^4^. Children have been shown to accumulate too much sitting time during their daily activities during and after school^5^. For example, a study conducted in the Republic of Ireland found that school-aged children spent approximately 61% of their waking time being sedentary (objectively measured by accelerometers)^6^. Another study in Brazil found that children spent most of their school time (64%) engaged in sedentary behavior (objectively measured by accelerometers)^7^. Alarmingly, the prevalence of sedentary time in children has intensified during the COVID-19 pandemic^8,9^. Thus, there have been several urgent calls to develop strategies to reduce children’s sedentary behavior.

Understanding the correlates of sedentary behavior among children is essential in developing effective interventions to reduce sitting time in this vulnerable population. Several previous studies have examined the correlates of domain-specific or objectively-measured sedentary behavior in children^10–14^. However, most previous studies focused on one or only a few levels of sedentary behavior correlates (i.e., personal, family, or school). Socioecological models of health behavior suggest that children’s sedentary behavior is influenced by personal, parental, organizational, and urban design factors^15,16^. Since these factors coexist and act together in daily life, it is necessary to consider them comprehensively in relation to children’s sedentary behavior. Additionally, correlates of sedentary behavior may differ across different sedentary behavior domains^16,17^. For example, while someone is reading this manuscript, their sitting time maybe is associated with having a chair-based desk^17^. Alternatively, if someone is sitting in a car to fulfil their daily activities, their car sitting time maybe is associated with the walkability of their residential neighbourhood^18,19^. These correlations may also depend on the societies in which they are examined because of different sociocultural norms. While studies exist in other geographical regions, there is a lack of evidence on the potential correlates of children’s sedentary behavior in Asia. Such evidence is vital in developing region-specific strategies to reduce sedentary behavior among children.

Therefore, this study aimed to identify correlates of domain-specific sedentary behaviors and objectively assessed sedentary time among a sample of children in Japan.

## Results

Data from 343 participants were analyzed, excluding those with missing variables (n = 141). Table 1 shows the characteristics of the study participants. The average age of the children was 8.8 years old (SD = 1.7). A total of 59.5% of the sample were female (n = 204), 91.5% had a healthy weight (n = 314), and 40.8% were from households with an annual household income ≥ ¥10,000,000 (n = 140).

**Table 1:** Characteristics of study participants (N = 343).

Variable	Mean (SD) or N (%)
Age (years)	8.8 (1.7)
Sex	
Female	204 (59.5)
Male	139 (40.5)
BMI	
Healthy weight	314 (91.5)
Overweight/Obese	29 (8.5)
Annual household income	
< ¥10,000,000	191 (55.7)
≥ ¥10,000,000	140 (40.8)
**Parental characteristics**	
Mother’s age	42.0 (4.8)
Mother’s educational level	
Tertiary or higher	209 (60.9)
Below tertiary	132 (38.5)
Mother’s employment status	
Full-time/part-time	219 (63.8)
Unemployed	121 (35.3)
Mother’s BMI	
Healthy weight	319 (93.0)
Overweight/Obese	14 (4.1)
Father’s age	44.0 (6.1)
Father’s educational level	
Tertiary or higher	242 (70.6)
Below tertiary	54 (15.7)
Father’s employment status	
Full-time/part-time	295 (86.0)
Unemployed	2 (0.6)
Father’s BMI	
Healthy weight	234 (68.2)
Overweight/Obese	63 (18.4)
**Household indoor environment**	
Number of TVs in the home	1.4 (0.7)
Having a video/DVD recorder/player	
No	13 (3.8)
Yes	329 (95.9)
Having a computer	
No	12 (3.5)
Yes	330 (96.2)
Having a video game console	
No	106 (30.9)
Yes	236 (68.8)
Having a TV in one’s own room	
No	213 (62.1)
Yes	7 (2.0)
**Residential neighborhood environment**	
Home’s Walk Score^®^	79.2 (14.5)
Safety	13.3 (3.0)
Pleasing aesthetics	11.8 (2.3)
Crime safety	5.0 (1.6)
Incivilities	2.8 (1.0)
**School environment**	
Equipment	9.4 (1.9)
Facilities	12.5 (2.7)
Safety	9.7 (1.6)
**School’s neighborhood environment**	
School’s Walk Score^®^	79.9 (9.8)

### Individual correlates models

Table 2 shows the individual regression models for social and environmental correlates of domain-specific sedentary behaviors and objectively assessed sedentary time among children. Of the 22 potential correlates, 14 were marginally significantly (*p* < 0.10) associated with different domain-specific sedentary behaviors and objectively assessed sedentary time (there are five, three, five, and one correlates from parental characteristics, household indoor environment, residential neighborhood environment, and school’s neighborhood environment, respectively). None of the school’s environment variables were marginally significant with any sedentary behaviors.

**Table 2:** Individual regression models for correlates of domain-specific sedentary behaviors and objectively assessed sedentary time among children.

	Reading or listening to music	TV or video viewing	Video game use	Computer use excluding class	Doing homework	Car riding	Smartphone use	Total sedentary time (Accelerometer-based)
	b (95% CI)	b (95% CI)	b (95% CI)	b (95% CI)	b (95% CI)	b (95% CI)	b (95% CI)	b (95% CI)
**Parental characteristics**								
Mother’s age	− 0.33 (− 7.82, 7.17)	3.30 (− 11.66, 18.27)	4.43 (− 2.76, 11.62)	**2.66 (**− **0.12, 5.44)**	− **10.14 (**− **22.19, 1.90)**	− **3.36 (**− **6.69, **− **0.04)**	− 4.52 (− 16.50, 7.46)	1.06 (− 0.82, 2.95)
Mother’s educational level								
Tertiary or higher	0	0	0	0	0	0	0	0
Below tertiary	− **63.96 (**− **118.53, **− **9.40)**	**95.55 (**− **13.37, 204.47)**	**47.85 (**− **4.49, 100.20)**	10.71 (− 9.50, 30.92)	− 11.28 (− 98.99, 76.42)	7.80 (− 16.39, 31.99)	**74.33 (**− **12.89, 161.55)**	− 9.03 (− 22.84, 4.78)
Mother’s employment status								
Full-time/part-time	0	0	0	0	0	0	0	0
Unemployed	0.55 (− 53.87, 54.98)	11.71 (− 96.92, 120.35)	− 27.17 (− 79.37, 25.04)	3.11 (− 17.04, 23.27)	57.32 (− 30.15, 144.79)	16.44 (− 7.69, 40.56)	10.27 (− 76.71, 97.26)	− 9.53 (− 23.21, 4.15)
Mother’s BMI								
Healthy weight	0	0	0	0	0	0	0	0
Overweight/Obese	− 64.50 (− 183.09, 54.09)	− 1.63 (− 238.34, 235.09)	5.37 (− 108.39, 119.13)	− 19.01 (− 62.93, 24.91)	− 54.40 (− 245.00, 136.20)	− 18.87 (− 71.44, 33.69)	− 34.52 (− 224.07, 155.03)	− 18.91 (− 48.78, 10.97)
Father’s age	− **7.20 (**− **13.90, **− **0.51)**	− 5.63 (− 18.99, 7.74)	− 3.21 (− 9.64, 3.21)	− 1.82 (− 4.30, 0.66)	**14.59 (3.83, 25.35)**	**2.97 (0.00, 5.94)**	8.36 (− 2.34, 19.07)	− 0.32 (− 2.02, 1.37)
Father’s educational level								
Tertiary or higher	0	0	0	0	0	0	0	0
Below tertiary	− 22.85 (− 94.32, 48.63)	**137.45 (**− **5.22, 280.12)**	− 21.49 (− 90.05, 47.07)	4.88 (− 21.60, 31.35)	− **109.13 (**− **224.01, 5.74)**	0.56 (− 31.12, 32.25)	− 16.26 (− 130.50, 97.99)	− **18.88 (**− **36.84, **− **0.91)**
Father’s employment status								
Full-time/part-time	0	0	0	0	0	0	0	0
Unemployed	208.33 (− 88.45, 505.11)	− 278.29 (− 870.68, 314.10)	− 5.24 (− 289.93, 279.45)	− 28.53 (− 138.44, 81.39)	102.68 (− 374.30, 579.67)	− 59.39 (− 190.95, 72.17)	− 73.56 (− 547.91, 400.79)	10.89 (− 63.71, 85.49)
Father’s BMI								
Healthy weight	0	0	0	0	0	0	0	0
Overweight/Obese	0.42 (− 64.24, 65.08)	− 23.04 (− 152.11, 106.02)	− **55.57 (**− **117.59, 6.46)**	− 9.83 (− 33.78, 14.12)	60.91 (− 43.01, 164.83)	3.76 (− 24.90, 32.43)	− 3.11 (− 106.46, 100.24)	0.43 (− 15.82, 16.68)
**Household indoor environment**								
Number of TVs in the home	− 35.84 (− 78.96, 7.29)	− 5.53 (− 83.19, 72.12)	14.48 (− 26.84, 55.79)	− 5.47 (− 26.09, 15.15)	15.18 (− 59.68, 90.05)	0.72 (− 17.64, 19.09)	− 14.59 (− 94.51, 65.34)	3.98 (− 7.74, 15.70)
Having a video/DVD recorder/player								
No	0	0	0	0	**0**	0	0	0
Yes	− 66.05 (− 216.12, 84.03)	20.86 (− 249.39, 291.10)	18.51 (− 125.28, 162.30)	18.53 (− 53.22, 90.28)	− **261.07 (**− **521.61, **− **0.53)**	13.40 (− 50.52, 77.32)	− 161.43 (− 439.58, 116.71)	5.30 (− 35.41, 46.02)
Having a computer								
No	0	0	0	0	**0**	0	0	0
Yes	− 2.75 (− 139.33, 133.82)	− 111.59 (− 357.51, 134.34)	− 36.57 (− 167.42, 94.28)	24.02 (− 41.27, 89.32)	146.00 (− 91.10, 383.10)	− 16.02 (− 74.19, 42.15)	96.48 (− 156.64, 349.60)	1.56 (− 35.57, 38.70)
Having a video game console								
*No*	0	0	0	0	**0**	0	0	0
*Yes*	31.25 (− 36.11, 98.61)	**225.09 (103.79, 346.39)**	**90.49 (25.95, 155.03)**	12.96 (− 19.24, 45.17)	− 29.19 (− 146.13, 87.76)	8.41 (− 20.28, 37.10)	**158.58 (33.73, 283.42)**	0.76 (− 17.56, 19.09)
Having a TV in one’s own room								
*No*	0	0	0	0	**0**	0	0	0
*Yes*	− 109.72 (− 268.87, 49.44)	**248.74 (**− **37.85, 535.32)**	**300.88 (148.40, 453.36)**	13.22 (− 62.87, 89.31)	− 5.91 (− 282.21, 270.39)	− 28.80 (− 96.59, 38.98)	**317.06 (22.10, 612.03)**	− 2.35 (− 45.63, 40.94)
**Residential neighborhood environment**								
Home’s Walk Score^®^	1.32 (− 0.33, 2.98)	− **4.29 (**− **7.61, **− **0.98)**	0.53 (− 1.17, 2.22)	**0.71 (0.00, 1.42)**	0.38 (− 2.15, 2.91)	− 0.54 (− 1.22, 0.15)	0.07 (− 2.66, 2.80)	0.10 (− 0.34, 0.53)
Safety	− 2.73 (− 10.83, 5.38)	3.14 (− 13.11, 19.39)	− 5.47 (− 13.76, 2.82)	1.62 (− 1.86, 5.09)	**12.35 (**− **0.05, 24.74)**	− **4.06 (**− **7.40, **− **0.71)**	− 5.10 (− 18.47, 8.26)	− 0.19 (− 2.33, 1.94)
Pleasing aesthetics	**11.10 (0.65, 21.56)**	− 16.17 (− 37.13, 4.79)	6.26 (− 4.43, 16.96)	− 1.68 (− 6.16, 2.80)	− 10.57 (− 26.56, 5.42)	2.97 (− 1.34, 7.29)	7.31 (− 9.94, 24.55)	− 0.35 (− 3.11, 2.41)
Crime safety	6.01 (− 9.25, 21.27)	− 12.69 (− 43.29, 17.90)	**15.03 (**− **0.57, 30.64)**	2.61 (− 3.93, 9.14)	2.17 (− 21.16, 25.51)	− 2.98 (− 9.28, 3.31)	− 17.01 (− 42.18, 8.16)	− 1.04 (− 5.08, 2.99)
Incivilities	**23.09 (0.03, 46.14)**	3.26 (− 42.96, 49.49)	− 11.07 (− 34.65, 12.52)	1.98 (− 7.89, 11.86)	− 22.10 (− 57.36, 13.16)	− 1.88 (− 11.39, 7.63)	20.67 (− 17.36, 58.70)	− 0.97 (− 7.07, 5.13)
**School environment**								
Equipment	11.84 (− 5.89, 29.57)	6.59 (− 28.71, 41.90)	− 2.61 (− 20.65, 15.42)	− 1.95 (− 9.54, 5.64)	17.29 (− 10.67, 45.26)	− 4.03 (− 11.25, 3.20)	− 1.98 (− 31.20, 27.24)	1.39 (− 3.21, 5.99)
Facilities	− 4.95 (− 18.77, 8.87)	− 8.21 (− 35.73, 19.31)	4.74 (− 9.32, 18.80)	− 0.63 (− 6.54, 5.29)	− 10.69 (− 32.49, 11.11)	− 0.05 (− 5.68, 5.59)	− 13.64 (− 36.41, 9.13)	− 2.59 (− 6.20, 1.03)
Safety	− 0.73 (− 21.25, 19.80)	− 4.83 (− 45.69, 36.03)	− 3.39 (− 24.27, 17.49)	4.69 (− 4.10, 13.47)	0.64 (− 31.73, 33.01)	2.32 (− 6.04, 10.69)	5.89 (− 27.93, 39.70)	1.95 (− 3.39, 7.29)
**School’s neighborhood environment**								
School’s Walk Score^®^	0.73 (− 2.04, 3.50)	− 0.46 (− 5.97, 5.05)	1.06 (− 1.76, 3.87)	0.32 (− 0.86, 1.51)	**4.19 (**− **0.15, 8.54)**	− 0.71 (− 1.84, 0.42)	− 0.20 (− 4.89, 4.48)	0.40 (− 0.33, 1.12)

*b* = unstandardized regression coefficients; *CI* Confidence interval. All models adjusted for children’s age, sex, BMI, annual household income, and locality. Bolded data indicate significance (*p *< 0.10).

### Fully adjusted correlates models

Table 3 presents the fully-adjusted regression models for correlates of domain-specific sedentary behaviors and objectively assessed sedentary time among children. Parental characteristics, including mother’s age and mother’s educational level, were negatively associated with daily minutes of doing homework (β = − 13.31, 95% CI: − 24.51, − 2.10, *p* = 0.02) and with daily minutes of reading or listening to music (β = − 66.97, 95% CI: − 118.52, − 15.43, *p* = 0.01), respectively. Father’s age was positively associated with daily minutes of doing homework (β = 15.62, 95% CI: 5.47, 25.78, *p* = 0.00). Among the household indoor environment variables, having a video/DVD recorder/player was negatively associated with daily minutes of doing homework (β = − 213.59, 95% CI: − 391.94, − 35.25, *p* = 0.02). Having a video game console at home was positively associated with daily minutes of TV or video viewing, video game use, and smartphone use (β = 225.15, 95% CI 108.27, 342.03, *p* = 0.00; β = 98.75, 95% CI: 32.26, 165.24, *p* = 0.00; and β = 144.06, 95% CI: 24.62, 263.51, *p* = 0.02, respectively). Having a TV in one’s own room was positively associated with daily minutes of video game use and smartphone use (β = 434.49, 95% CI: 257.59, 611.39, *p* = 0.00; β = 301.08, 95% CI 17.66, 584.71, *p* = 0.04, respectively). Among residential neighborhood variables, home’s Walk Score^®^ was positively associated with daily minutes of computer use excluding classes (β = 0.78, 95% CI: 0.07, 1.49, *p* = 0.03). Safety was also negatively associated with daily minutes of car riding (β = − 4.43, 95% CI: − 8.34, − 0.053, *p* = 0.03). No significant associations were observed between the school’s neighborhood environment (measured by Walk Score^®^) and sedentary behaviors. There were also no significant associations between potential correlates and accelerometer-based total sedentary time.

**Table 3:** Fully adjusted regression models for correlates of domain− specific sedentary behaviors and objectively assessed sedentary time among children.

	Reading or listening to music	TV or video viewing	Video game use	Computer use excluding class	Doing homework	Car riding	Smartphone use	Total sedentary time (Accelerometer-based)
	b (95% CI)	b (95% CI)	b (95% CI)	b (95% CI)	b (95% CI)	b (95% CI)	b (95% CI)	b (95% CI)
**Parental characteristics**								
Mother’s age	–	–	–	0.62 (− 1.55, 2.79)	− **13.31 (**− **24.51, **− **2.10)**	− 2.78 (− 5.81, 0.25)	–	–
Mother’s educational level								
Tertiary or higher	0	0	0	–	–	–	0	–
Below tertiary	− **66.97 (**− **118.52, **− **15.43)**	62.85 (− 45.64, 171.34)	13.31 (− 47.14, 73.75)	–	–	–	22.31 (− 84.83, 129.45)	–
Father’s age	− 4.32 (− 8.65, 0.02)	–	–	–	**15.62 (5.47, 25.78)**	2.25 (− 0.16, 4.65)	–	–
Father’s educational level								
Tertiary or higher	–	0	–		0	–	–	0
Below tertiary	–	115.29 (− 21.14, 251.72)	–	–	− 81.66 (− 184.90, 21.59)	–	–	− 16.48 (− 33.59, 0.62)
Father’s BMI								
Healthy weight	–	–	0					
Overweight/Obese			− 37.08 (− 106.70, 32.54)	–				
**Household indoor environment**								
Having a video/DVD recorder/player								
No	–	–	–	–	0	–	–	–
Yes	–	–	–	–	− **213.59 (**− **391.94, **− **35.25)**	–	–	–
Having a video game console								
No	–	0	0	–	–	–	0	–
Yes	–	**225.15 (108.27, 342.03)**	**98.75 (32.26, 165.24)**	–	–	–	**144.06 (24.62, 263.51)**	–
Having a TV in one’s own room								
No	–	0	0	–	–	–	0	–
Yes	–	− 44.49 (− 355.33, 266.35)	**434.49 (257.59, 611.39)**	–	–	–	**301.18 (17.66, 584.71)**	–
**Residential neighborhood environment**								
Home’s Walk Score^®^	–	− 2.54 (− 6.31, 1.23)	–	**0.78 (0.07, 1.49)**	–	–	–	–
Safety	–	–	–	–	9.73 (− 3.99, 23.44)	− **4.43 (**− **8.34, **− **0.53)**	–	–
Pleasing aesthetics	5.33 (− 5.78, 16.44)	–	–	–	–	–	–	–
Crime safety	–	–	4.91 (− 14.83, 24.65)	–	–	–	–	–
Incivilities	23.40 (− 2.34, 49.13)	–	–	–	–	–	–	–
**School’s neighborhood environment**								
School’s Walk Score^®^	–	–	–	–	4.85 (0.00, 9.70)	–	–	–

b = unstandardized regression coefficients; CI = confidence interval. All models adjusted for children’s age, sex, BMI, annual household income, and locality. Bolded data indicate significance (*p *< 0.05).

## Discussion

The present study is one of the few studies that has explored the correlates of domain-specific sedentary behaviors and objectively assessed sedentary time among children in the less-studied geographical context of Asia. Consistent with some previous studies^20–22^, several parental characteristics were associated with children’s sedentary time in different contexts. Mothers with a lower education level was associated with their child spending more time reading or listening to music. Mother’s and father’s age were associated with their child spending lower and higher time devoted to doing their homework, respectively. There may be several mechanisms, such as parental support, shared activities, and societal differences by generation, through which these parental characteristics may impact children's sedentary time^23–25^. For example, a study conducted among 6^th^-grade children in the United States found that girls with higher parental support were more likely to be less sedentary after school-time^26^. While some parental characteristics are mainly nonmodifiable (or difficult to change), they can provide helpful socioeconomic information on public health programs to reduce children’s sedentary lifestyles. Further studies are needed to develop successful programs varying by different parental characteristics. This study also identified several household indoor environment correlates of children’s sedentary behavior. The availability of TV, DVD players, and video game consoles in the household was associated with screen-based sedentary behaviors in children. These findings are consistent with several previous studies^11,20,27,28^. For instance, a study conducted in Canada found that the number of TV sets and video game device availability were positively associated with children’s screen time^28^. Another study in the USA found that fewer media devices in the bedroom were associated with lower sedentary behavior in the children^11^. Notably, having a video/DVD/recorder player was negatively associated with doing homework in our study. This result aligns with the evidence that the availability of screen-based devices such as a TV and video game device in the home may negatively impact academic performance^29,30^. These findings suggest that interventions are needed to encourage children to have less screen time (e.g., educating parents and children about the importance of limiting screen time encouraging households to remove TVs from bedrooms).

Our findings showed that residential neighborhood’s perceived pedestrian and cyclist safety was negatively associated with children’s car riding. This finding supports some previous studies showing that a safe neighborhood for walking and cycling can discourage travel by car^31,32^. Safety from traffic has been identified as one of the critical barriers to children’s active travel, such as walking and cycling^33,34^. Parents are generally reluctant to allow their children to walk or cycle if their neighborhood is perceived as unsafe^35,36^. For instance, a study in the USA found that parents’ better perceptions of traffic safety were associated with children’s independent mobility (i.e., walking, cycling, public transport)^36^. Our findings suggest that interventions that improve neighborhood pedestrian and cyclist safety perceptions may reduce children’s time spent riding in cars. There was also a positive association between home’s Walk Score^®^ and computer use in our sample. The exact reasons for this positive association remain to be elucidated. Some unmeasured variables may explain this association (i.e., households in a walkable neighborhood may have more computer devices).

We did not find significant associations between accelerometer-based sedentary time and potential correlates. There may be several reasons for these findings. The main reason is that objective sedentary time was not context-specific, whereas several sedentary behaviors are more likely to be undertaken in specific contexts that align with the correlates being examined (e.g., household environments). This suggests that the total accelerometer-based sedentary behavior examined here was less likely to be undertaken in those contexts. This is also supported by fewer associations observed for neighborhood and school environments in our study. Additionally, most households in Japan have some type of screen-based device. For example, the proportion of households in Japan with TVs and game consoles was 96.2% and 90.4%, respectively^37^. Even those households that do not have a TV in the bedroom likely have a computer or TV elsewhere in the house. These findings support the evidence that sedentary behavior is a context-specific behavior, and initiatives to reduce it should consider this attribute^16,17^. Together, these findings suggest that while interventions targeting residential and home environments might reduce sedentary behavior, they may not reduce the overall sedentary time (as measured by accelerometers). The children were attending school during the period that the data were collected. The correlates examined were not associated with accelerometer-measured sedentary time; therefore, this finding might suggest that much of this sedentary time is being accumulated at school. This might indicate that school-based interventions designed to reduce prolonged sitting might be required.

This study has some limitations. As a cross-sectional study, causal relationships between variables cannot be inferred. The perceived measures of home and neighborhood factors may be subject to bias. In addition, using a questionnaire to extrapolate time in domain-specific sedentary behaviors introduces recall bias. Similar to previous studies on children, the survey was completed by parents with children. There may be a disagreement between parents’ and children’s perceptions of the environment. We could also not account for simultaneously participating in multiple sedentary behaviors (e.g., riding in the car while using a smartphone). Our accelerometer-derived sedentary time was only available across the entire day. Future studies must collect accelerometer data separately during school and leisure time. The only main effects were estimated in our study however, it is possible that interactions exist between the correlates and future research should explore this possibility. Furthermore, while a comprehensive list of potential correlates was included in this study, there are still missing factors such as cell phones and tablets, parks and playgrounds that may get children out of the house and therefore engaged in less sedentary time. Another limitation is that the household indoor environment variables only reflected those supportive of sedentary behavior. Other household indoor environment variables that discourage sedentary time were not considered in the analysis (e.g., owning a dog or pet, having a backyard, having siblings, owning a bicycle, or not owning a motor vehicle). Furthermore, our sample had a higher household income level than the average population. Notably, the percentage of households in Japan receiving 10 million JPY is approximately 12.1%, according to a national survey in 2019^38^. The strengths of this study were using domain-specific sedentary behavior, objectively-assessed sedentary time, exploring the correlates from a socioecological perspective, and focusing on a less-explored setting in Asia.

## Conclusions

The present study contributed to the limited (but fast-growing) body of research on the correlates of sedentary behavior in children. Focusing on a less-explored setting and population in Asia, we found several correlates for children’s sedentary behavior. These correlates differed across different domains of sedentary behavior. In summary, mother’s age and education were negatively associated with children’s reading or listening to music and doing homework, respectively. Father’s age was associated with children spending more time doing their homework. Living in neighborhoods with higher perceived pedestrian and cyclist safety was associated with lower time spent in the car riding among children. Home’s Walk Score^®^ was associated with children’s higher computer use (excluding classes). Children with TV, DVD players, and video game consoles in their household were more likely to spend time in screen-based sedentary behvaiors. Future studies in different geographical contexts are needed to inform local urban design and public health policies to reduce sedentary time among children.

## Methods

### Data source and participants

**Figure 1:**
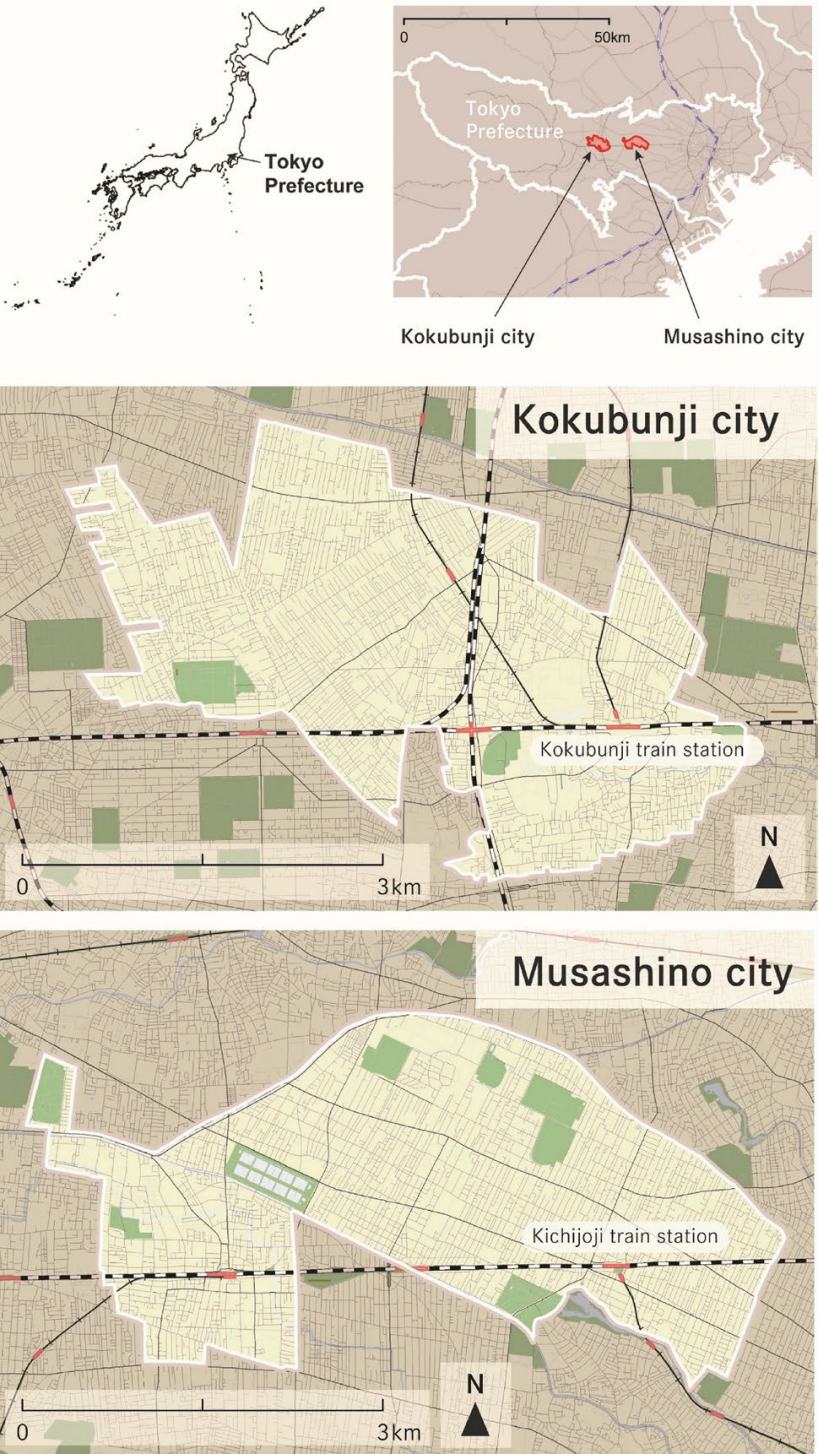
The locations of Musashino city and Kokubunji city in Japan.

This study included cross-sectional data from a cohort of children living in Japan. Data were obtained between February and March 2017 and September and October 2018, a period during which children were attending school, from a randomly selected sample of residents living in two Japanese urban localities: Musashino city (150,660 persons in 2021) and Kokubunji city (130,636 persons in 2021) (Fig. 1).

**Figure 2:**
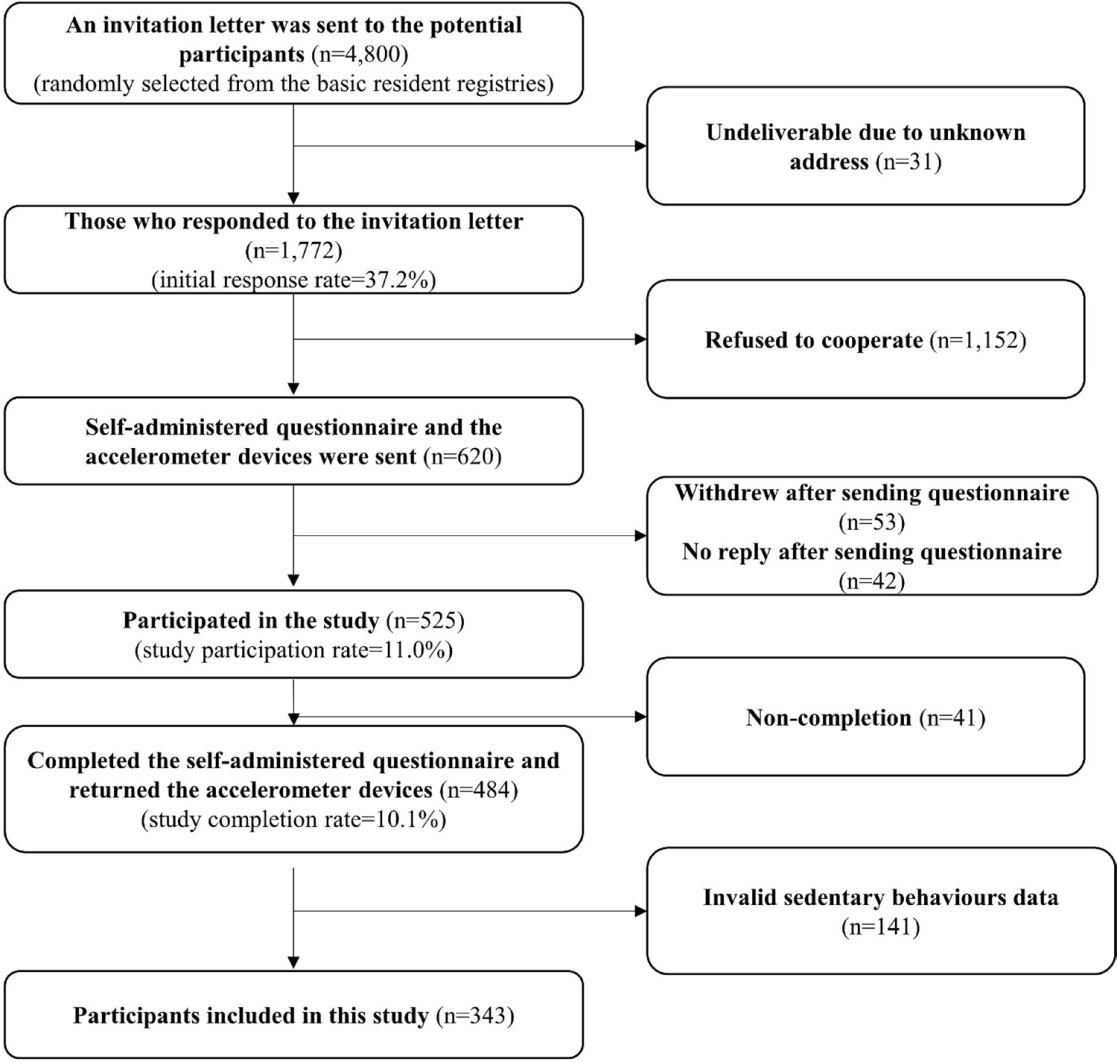
Flow diagram of participants in the study.

An invitation letter was sent to 4,800 children sampled from the government basic resident register and stratified by sex (boys/girls), school grade (1st grade: 6–7 years, 2nd grade: 7–8 years, 3rd grade: 8–9 years, 4th grade: 9–10 years, 5th grade: 10–11 years, and 6th grade: 11–12 years). An exact number of children was selected from each urban locality (Musashino City/Kokubunji City). A reminder letter was sent to nonrespondents two weeks after the initial mailing. A total of 1,772 participants responded to the invitation letter expressing their interest in participation in this study (initial response rate = 37.2%). A self-administered questionnaire, an accelerometer, and a consent form were mailed to a total of 620 households who finally agreed to participate in the study (1,152 households refused to cooperate). Of these, 484 households (one child in each household) completed the questionnaire and returned the accelerometers (Fig. 2). A 1,000-yen (equivalent to approximately US$10) book voucher was offered to those who returned the completed questionnaire and accelerometer. Since children may be unable to accurately report their activity patterns^39^, parents (including caregivers) completed the questionnaire with their children. Both parents and children provided written informed consent prior to participation. The study was approved by the Research Ethics Committee, Waseda University, Japan (2017–245) and the methods were carried out in accordance with these guidelines.

### Measures

#### Outcome variables

##### Domain-specific sedentary behaviors

Domain-specific leisure-time sedentary behavior was divided into the following seven domains using a validated Japanese questionnaire^40^: (1) reading or listening to music, (2) TV or video viewing, (3) video game use, (4) computer use (outside of class), (5) doing homework, (6) car riding, and (7) smartphone use. The participants reported how many days (a continuous number) on average per week (in a usual week) and how much time (hours and minutes, continuous numbers) on average per day they engaged in these sedentary behaviors during weekdays and weekends. The average daily minutes of each domain’s sedentary time was calculated with weighting to account for the number of weekdays and weekend days.

##### Objectively assessed sedentary time

A validated triaxial accelerometer (Active style Pro, HJA-750C; Omron Healthcare, Kyoto, Japan) was used to assess sedentary time for seven consecutive days^41,42^. The data were collected in 10-s epochs and expressed as metabolic equivalents (METs). When the Active style Pro device is used to evaluate sedentary behavior and physical activity in primary school children, the values of METs are overestimated^43^. Therefore, we used the following conversion equations for primary school children obtained from the results of Hikihara et al.^43^:

Ambulatory activities: 0.6237 × MET value of Active style Pro + 0.2411.

Nonambulatory activities: 0.6145 × MET value of Active style Pro + 0.5573.

The participants were instructed to wear the accelerometer on their hip throughout the day for at least seven consecutive days except when sleeping or during water-related activities (e.g., bathing, swimming) or contact sports (e.g., soccer or rugby). Nonwear time was defined as intervals of at least 20 consecutive min of 0 METs, and the recording was considered valid when the device was worn for at least 10 h per day^44^. Those who wore the accelerometer for a minimum of 4 days (including at least one weekend day) and at least 10 h per day were included in this study^44,45^. The daily average time spent on sedentary time was calculated (≤ 1.5 METs)^4,43,44,46^.

### Potential correlates

#### Parental characteristics

Both parents (or a single parent) were asked to report their age, educational level (tertiary or higher versus below tertiary), employment status (full-time/part-time versus unemployed), weight, and height. Body mass index (BMI) was calculated from the height and weight data (weight/height^2^). The parents were classified based on their BMI as healthy weight (< 25 kg/m^2^) and overweight/obese (≥ 25 kg/m^2^).

#### Household indoor environment

The household environment was assessed using a validated self-report questionnaire^47^. The parents and children reported their household indoor environment using the following items: number of TVs in the home; having a video/DVD recorder/player (no/yes); having a computer (no/yes); having a video game console (no/yes), and having a TV in one’s own room (no/yes).

#### Residential neighborhood environment

Objective and perceived measures of participants’ residential neighborhood environment were included. Walk Score^®^, an objective measure of neighborhood walkability, was obtained for each participant’s residential address from their website (www.walkscore.com). Walk Score^®^ is a publicly-available measure that assigns a walkability score (low = 0 to high = 100) to any given address based on access to various destinations, residential density, and street connectivity^48^. Walk Score^®^ is a valid indicator of neighborhood walkability in the Japanese context^49^. A higher Walk Score^®^ indicates that a location is more supportive of walking^48^, including for children^50^. A study conducted in Japan found that a higher Walk Score^®^ was associated with adults’ walking and negatively associated with car driving^51^. The perceived residential neighborhood environment, including safety, pleasing aesthetics, crime safety, and incivilities, was assessed using a 13-item questionnaire developed in Japanese contexts^52^. The parents read the questionnaire to their children and recorded their answers. This questionnaire had acceptable construct validity (factor analysis, maximum-likelihood factor loading: safety = 0.58–0.81, pleasing aesthetics = 0.46–0.91, crime safety = 0.67–0.77, incivilities = 0.68, 0.69), and test–retest reliability (safety, r = 0.65; pleasing aesthetics, r = 0.68; crime safety, r = 0.55; incivilities r = 0.57). The safety factor consisted of five items: “the streets in my neighborhood are safe”, “it is safe to walk or ride a bicycle to school”, “the area is safe to walk or ride a bicycle”, “intersections are safe”, and “it is easy to walk or ride a bicycle”. The pleasing aesthetics factor included four items: “my neighborhood has a nice yard (attractive yard)”, “there are many nice houses (attractive houses) in the neighborhood”, “the neighborhood is a nice and quiet place”, and “there is a lot of nature in the neighborhood”. The crime safety factor had two items: “I'm worried about suspicious people”, “I'm worried about the bad guys following me around”. The incivilities factor had two items: “there is a lot of graffiti in the neighborhood” and “there is a lot of garbage in the neighborhood”. The participants chose a response on a 4-point scale (1: “strongly disagree” to 4: “strongly agree”). Each factor was scored by summing the answers.

#### School environment

The school environment was measured using a validated self-report questionnaire^52^. The scale comprised 10 items representing three factors: equipment, facility, and safety. The scale had acceptable construct validity (factor analysis, maximum-likelihood factor loading: equipment = 0.62–0.90, facility = 0.51–0.92, safety = 0.57–0.84), internal consistency (equipment, α = 0.87; facility, α = 0.84; safety, α = 0.86) and test–retest reliability (equipment, r = 0.52; facility, r = 0.68; safety, r = 0.51). The equipment factor consisted of three items: “the equipment at my school is easy to use for engaging physical activity and sports (e.g., horizontal bar and ball), “my school has enough equipment that I can use actively,” and “my school has enough equipment that I can use actively during the recess period.” The facility factor included four items: “my school’s grounds are easy to use,” “my school’s gym is easy to use,” “my school’s grounds are large enough to allow me to be active,” and “my school’s gym is large enough so that I can spend time there being active.” The safety factor had three items: “the grounds and gym at my school are safe to use,” “the school facilities are safe to use for engaging physical activity,” and “my school’s gym and grounds are well-maintained.” The participants chose a response on a 4-point scale (1: “strongly disagree” to 4: “strongly agree”). Responses were scored for each factor by summing the answers.

#### School’s neighborhood environment

Walk Score^®^ was used to measure the school’s neighborhood environment. A Walk Score^®^ was assigned to each school based on that school’s address.

### Covariates

Children’s age and sex (female versus male) were obtained from the government residential registries. Children’s weight and height were obtained from the questionnaire reported by their parents. BMI was calculated from the height and weight data (weight/height^2^). The children were classified on the basis of their BMI as healthy weight (< 85th percentiles) and overweight/obese (≥ 85th to < 95th percentiles)^53^. Parents reported their annual household gross income (< ¥10,000,000 versus ≥ ¥10,000,000). These covariates were selected because of their potential associations with children’s active and sedentary behaviors^54,55^.

### Statistical analysis

Descriptive information (i.e., means and standard deviations) was estimated for children’s sociodemographic, social and environmental, and sedentary behavior variables. Multivariable linear regression models were used to identify the correlates of objectively assessed sedentary time and domain-specific sedentary behaviors. The models included a dichotomous area variable Musashino city = 0 and Kokubunji city = 1) as a covariate representing locality effects at the city level. Each potential correlate was first included in individual regression models with each of the outcomes. Variables that were marginally significant (*p* < 0.10) were then included in the fully adjusted regression models. All models included age, sex, BMI, and annual household income as covariates. Multicollinearity of variables included in the models was tested using the variance inflation factor (VIF), and no issue of multicollinearity was detected (VIF < 5)^56^. For all point estimates (*b* = unstandardized regression coefficients), 95% confidence intervals (CIs) were estimated. Analyses were conducted using Stata 15.0 (Stata Corp, College Station, Texas), and the significance level was set at *p* < 0.05.

## Data Availability

The datasets generated and/or analysed during the current study are not publicly available due to ethical and legal constraints but anonymized data are available from the corresponding author on reasonable request.
